# Differentiation between genomic and non-genomic feedback controls yields an HPA axis model featuring Hypercortisolism as an irreversible bistable switch

**DOI:** 10.1186/1742-4682-10-65

**Published:** 2013-11-09

**Authors:** Clemens A Zarzer, Martin G Puchinger, Gottfried Köhler, Philipp Kügler

**Affiliations:** 1 Institute for Applied Mathematics and Statistics, University of Hohenheim, Schloss, 70599 Stuttgart, Germany; 2 RICAM, Austrian Academy of Sciences, Altenbergerstrasse 69, 4040 Linz, Austria; 3 Max F. Perutz Laboratories, Dr. Bohr-Gasse 9, 1030 Vienna, Austria

## Abstract

**Background:**

The hypothalamic-pituitary-adrenal axis (HPA axis) is a major part of the neuroendocrine system responsible for the regulation of the response to physical or mental stress and for the control of the synthesis of the stress hormone cortisol. Dysfunctions of the HPA axis characterized by either low (hypocortisolism) or increased (hypercortisolism) cortisol levels are implicated in various pathological conditions. Their understanding and therapeutic correction may be supported by mathematical modeling and simulation of the HPA axis.

**Methods:**

Mass action and Michaelis Menten enzyme kinetics were used to provide a mechanistic description of the feedback mechanisms within the pituitary gland cells by which cortisol inhibits its own production. A separation of the nucleus from the cytoplasm by compartments enabled a differentiation between slow genomic and fast non-genomic processes. The model in parts was trained against time resolved ACTH stress response data from an *in vitro* cell culture of murine AtT-20 pituitary tumor cells and analyzed by bifurcation discovery tools.

**Results:**

A recently found pituitary gland cell membrane receptor that mediates rapid non-genomic actions of glucocorticoids has been incorporated into our model of the HPA axis. As a consequence of the distinction between genomic and non-genomic feedback processes our model possesses an extended dynamic repertoire in comparison to existing HPA models. In particular, our model exhibits limit cycle oscillations and bistable behavior associated to hypocortisolism but also features a (second) bistable switch which captures irreversible transitions in hypercortisolism to elevated cortisol levels.

**Conclusions:**

Model predictive control and inverse bifurcation analysis have been previously applied in the simulation-based design of therapeutic strategies for the correction of hypocortisolism. Given the HPA model extension presented in this paper, these techniques may also be used in the study of hypercortisolism. As an example, we show how sparsity enforcing penalization may suggest network interventions that allow the return from elevated cortisol levels back to nominal ones.

## Background

The HPA axis is a major part of the neuroendocrine system in mammals and particularly in humans. A main task of this hormonal network is the regulation of the response to physical or mental stress that threatens to disrupt the homeostatic balance of the organism. If a stressor is sensed by the nervous system the hypothalamus is stimulated to produce and secret the corticotropin-releasing hormone (CRH). The secretion of CRH causes the anterior pituitary to synthesize adrenocorticotropin (ACTH). ACTH then stimulates the adrenal glands to release cortisol, which down regulates the blood concentration of CRH and ACTH via different negative feedback mechanisms [1-4].

The HPA axis is subject of intensive research in endocrinology as HPA malfunctions are implicated in various pathological conditions. These are often characterized by either elevated or insufficient blood cortisol levels compared to the average healthy human. For instance, hypocortisolism (insufficient cortisol level) is reported in patients suffering from the chronic fatigue syndrome and post traumatic stress disorders (cf. [5-9]), whereas hypercortisolism (elevated cortisol level) is observed in depression, dementia or postoperative delirium (cf. [10-15]). Especially in the context of personalized medicine the use of modeling and simulation of biological systems for the rational design of treatments and drug intervention strategies is more and more recognized [16-20]. For such endeavors the integration of biological information of different type into computational, hence predictive, models is a prerequisite. The emphasis of earlier HPA modeling approaches with ordinary and delay differential equations has been put on self regulatory ultradian and circadian oscillatory behavior in [21-27], oscillations in response to an independent external pacemaker from the suprachiazmatic nucleus have been described in [28,29]. In comparison, the article [30] stands out as it incorporates intracellular glucocorticoid receptor kinetics which mediate bistable behavior of the HPA axis. Despite its parsimonious character the four state ODE model of [30] offers an explanation for hypocortisolism as an irreversible biological switch and served in [31] as a basis for the design of a therapeutic corrections of the HPA dysfunction. In [19] it is shown that the model of [30] also exhibits stable limit cycle oscillations, in [32] the four state rate equations of [30] were modified in order to fit oscillatory data of patients suffering from post traumatic stress disorder.

### A parsimonious HPA model featuring hypocortisolism

**Figure 1:**
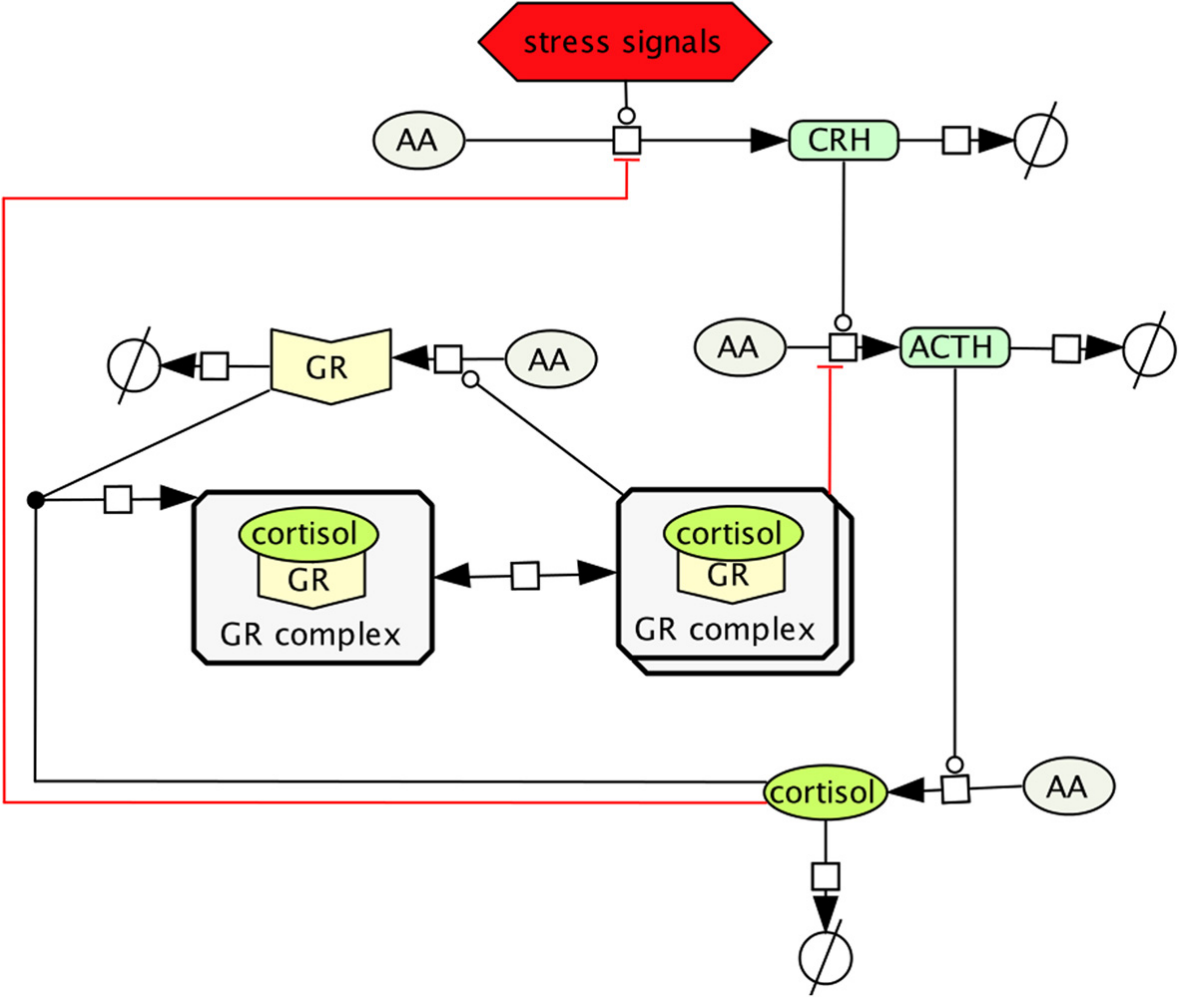
**The HPA axis feedback network.** Diagram of the biochemical feedback network of the HPA axis as modeled in [30]. Physical or mental stress triggers the secretion of corticotropin releasing hormone CRH in the hypothalamus. CRH is transported to the anterior pituitary gland and stimulates the release of the adrenocorticotropic hormone ACTH. ACTH signals to the adrenal gland to secrete cortisol COR. After binding to the glucocorticoid receptor GR in the pituitary, cortisol negatively regulates the production of CRH and ACTH. The homo dimerization introduces a positive feedback loop giving rise to bistable behavior in accordance with hypocortisolism.

The HPA axis model of [30] captures the basic feedback mechanisms and includes an intracellular glucocorticoid receptor GR in the anterior pituitary gland as one of the four state variables, see Figure 1. The dynamics of the model are described by the ODE system

(1) $$\begin{array}{l} \;\frac{d[\text{CRH}]}{dt}=\frac{k_{c}+\text{stress}}{1+\frac{\left[\text{COR}\right]}{k_{i1}}}-k_{\text{cd}}[\text{CRH}], \end{array}$$

(2) $$\begin{array}{l} \frac{d[\text{ACTH}]}{dt}=\frac{k_{a}[\text{CRH}]}{1+\frac{\left[\text{COR}-\text{GR}\right]}{k_{i2}}}-k_{\text{ad}}[\text{ACTH}], \end{array}$$

(3) $$\begin{array}{l} \;\frac{d[\text{GR}]}{dt}=\frac{k_{r}\;{\left[\text{COR}-\text{GR}\right]}^{2}}{k+{\left[\text{COR}-\text{GR}\right]}^{2}}+k_{\text{cr}}-k_{\text{rd}}[\text{GR}], \end{array}$$

(4) $$\begin{array}{l} \;\frac{d[\text{COR}]}{dt}=k_{o}[\text{ACTH}]-k_{\text{od}}[\text{COR}], \end{array}$$

where the ODE variables [*CRH*], [*ACTH*], [*GR*] and [*COR*] denote the concentrations of CRH, ACTH, GR and cortisol. If GR is bound to cortisol, the resulting complex is denoted by [*COR-GR*]. As this binding is very fast compared to the other dynamics it is assumed to be in equilibrium, i.e. [*COR-GR*] = [*COR*] · [*GR*]. The GR dimerization may generate an irreversible bistable switch in the model by which the dysfunctional abidance of the system at an alternative stable steady state characterized by low cortisol level can be explained. The therapeutic strategy of [31] for the correction of hypocortisolism based on (1)-(4) is to force the HPA axis by a further suppression of cortisol to a point where the only stable condition in proximity is the original healthy state. An alternative for supporting the search for pharmaceutical therapies is the manipulation of the feedback network by means of inverse bifurcation techniques in order to transform the irreversible switch into a reversible one, see [19,33]. Though the model of [30] sheds new light on possible causes for hypocortisolism, its dynamic repertoire could not be shown to feature the opposite scenario of hypercortisolism. Furthermore, while the model takes the intracellular glucocorticoid receptor GR into account, it does not consider feedback mechanisms known to act on faster time scales.

### Motivation of model extensions

The above mentioned GR mediated negative feedback on the production of ACTH acts at the genomic level. Specifically, cortisol COR binds to the glucocorticoid receptor GR and forms a homodimer (cf. [34,35]). This homodimer is known to work as a transcription factor, enhances the expression of the GR gene and down regulates the expression of the ACTH related gene. A consequence of the transcriptional regulation is that the GR feedback control works rather slowly compared to other cellular processes such as a vesicle transport, cell signaling or extracellular events such as changes in the CRH blood concentration. For mammalian cells one can expect at least a delay of the down regulation in the range of 15 minutes up to 2 hours (c.f. [1]). However, it has been frequently reported that cortisol induced inhibitory effects on anterior pituitary gland cells can already occur after a few seconds (c.f. [36,37]), a phenomenon that cannot be explained by means of the genomic feedback mechanism.

The common hypothesis for this observation is that besides the GR receptor a second receptor in the membrane may directly inhibit the release of ACTH when bound to cortisol (cf. [38-42]). In [43] the authors were able to provide evidence of a glucocorticoid receptor (GPCR) in the anterior pituitary cell membrane, possibly enabling the fast response related to cortisol induced inhibition. In the following we incorporate this fast and non-genomic feedback mechanism and for this purpose integrate a detailed pituitary gland cell model into the HPA network of [30]. As a consequence, the extended dynamic repertoire of the resulting HPA axis feedback network also features bistable behavior compatible with the dysfunctional state of hypercortisolism and the ability to simulate time resolved ACTH stress response data from *in vitro* AtT-20 pituitary tumor cells.

## Results

**Figure 2:**
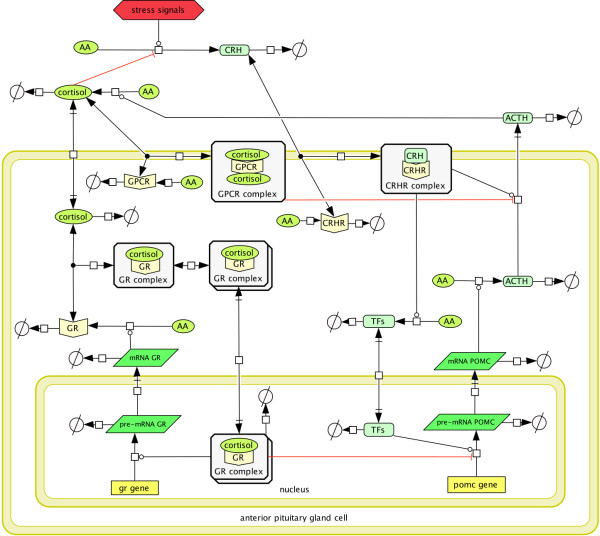
**Integration of an anterior pituitary gland cell model into the HPA network.** Diagram of the biochemical feedback network of the HPA axis after incorporation of the cortisol mediated feedback mechanisms in the anterior pituitary gland cells. The recently found membrane glucocorticoid receptor GPCR [43] and the intracellular receptor GR introduced in [30] act on different time scales. The modeling of the corresponding feedback mechanisms requires a distinction between slow genomic processes within the nucleus and fast non-genomic processes within the cytoplasm. In particular, the dimerized cortisol-glucocorticoid receptor complex (GR complex) and the transcription factors TF associated to the CRH receptor CRHR are explicitly considered. See Figure 3 for further details.

Using mass action and Michaelis Menten enzyme kinetics we developed a mechanistic ODE system model of the glucocorticoid feedback mechanisms within the anterior pituitary gland cell. In particular, we incorporated the glucocorticoid membrane receptor GPCR found in [43] and distinguished between slow genomic and fast non-genomic processes by using different compartments for the nucleus and the cytoplasm. Figure 2 displays the reaction network graph of the resulting HPA axis model while the details are discussed in the Methods section.

**Figure 3:**
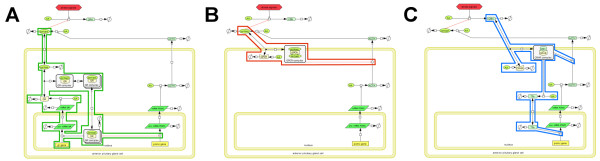
**The three core feedback pathways of the (anterior) pituitary gland cell model.** Illustration of the intracellular pathways of the extended HPA model of Figure 2 showing the three main feedback mechanisms (with respect to the involved receptors) in the anterior pituitary gland cells. **A)** Genomic pathway of the GR mediated feedback: Outline of the modeled species involved in the inhibition of POMC (Proopiomelanocortin) transcription which corresponds to the inhibition of ACTH production. ACTH is obtained via post-translational cleavaging of the POMC protein. The central transcription factor is the GR-cortisol complex which down regulates the POMC transcription and enhances the production of the GR receptor. **B)** Non-genomic pathway of the GPCR mediated feedback: The non-genomic negative feedback mechanism via the glucocorticoid membrane receptor GPCR, inhibiting the release of ACTH. **C)** CRHR mediated feedback: The positive feedback mechanisms associated with the CRH membrane receptor (CRHR), which causes an increased release of ACTH from vesicles and an increased expression of the POMC gene.

### Model comparison with experimental data

**Figure 4:**
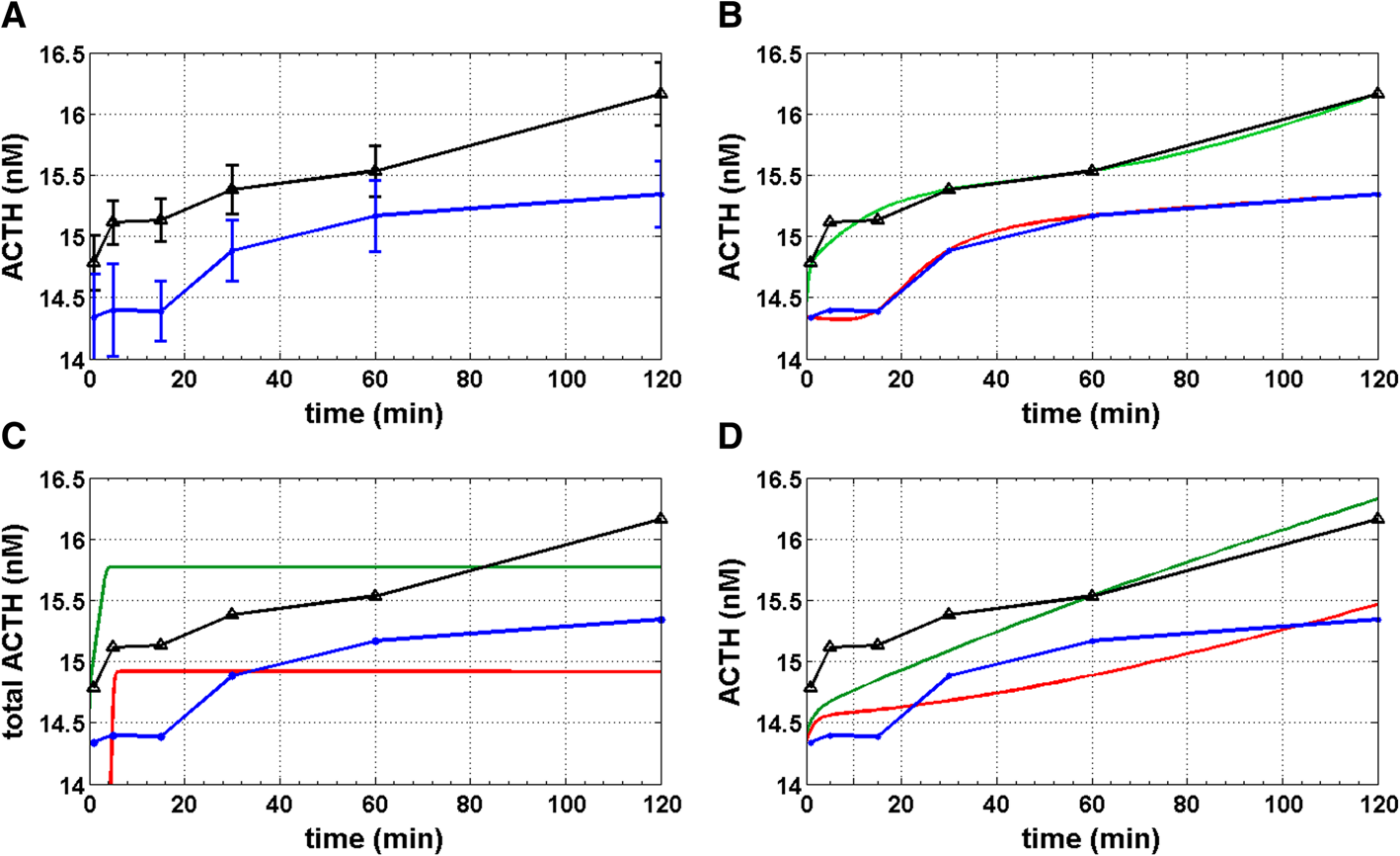
**Experimental and simulated ACTH concentration profiles.** The figure shows experimental ACTH profiles (black and blue lines) from murine AtT-20 pituitary cell lines described in [44] and simulations (green and red lines) after data fitting with different HPA network models. **A)** The black line corresponds to an experiment in which the system was exposed to 10 nM of CRH at time zero, the blue line corresponds to an experiment in which - in addition to 10 nM - 100 nM of cortisol was administered at time zero. The error bars show the 95% confidence intervals for the measurements at the different time points. **B)** Fit of experimental data with the HPA network model of Figure 2. **C)** Fit of experimental data with the HPA network model of Figure 2 after knockout of the membrane glucocorticoid receptor GPCR pathway. **D)** Fit of experimental data with the HPA network model of Figure 1. The results underpin the need for including the membrane glucocorticoid receptor GPCR in models of the pituitary gland cells.

The experimentally observed fast inhibitory effect of cortisol on the anterior pituitary gland cell motivated the model incorporation of the GPCR receptor in order to allow for non-genomic, hence fast, signal processing. In order to test the capability of our model to capture these fast effects against actual data, we conducted experiments in cultures of the murine AtT-20 pituitary cell line (cf. [44]). After administering doses of CRH and cortisol samples were taken from the medium and analyzed with respect to the total ACTH concentration in the cell medium, see Figure 4A for the data obtained for two different experimental setups. In the first setup (indicated by the triangle markers in Figure 4A) we considered the situation of a single stress stimulus by 10nM CRH at time point zero in the absence of cortisol. We observed an immediate increase of the ACTH secretion, followed by a slight stagnation and then a constantly increasing total ACTH concentration. In the second experiment (indicated by the dot markers in Figure 4A) 10nM CRH and in addition 100nM cortisol were administered. As depicted, this immediately activated the fast non-genomic pathway inhibiting the release of ACTH. This led to an increased amount of ACTH stored in the vesicles within the cell and thus to a delayed but strong release of ACTH after approximately 15 minutes. However, after 30 to 60 minutes the genomic feedback mechanisms become effective resulting in an overall reduced ACTH concentration in the medium.

For training the model against the experimental data by adaptation of the model parameters we followed a variational regularization approach (cf. [33,45]), see Methods for details. Sparsity enforcing penalization was used to eliminate model components less relevant for reproducing the observed kinetics. Figure 4B shows that the experimental data can be fitted by the HPA model of Figure 2 even if the autocatalytic loop for the GR gene regulation would be neglected and if the production of the TF (transcription factors for CRH) receptors would be considered to be independent of CRH.

In order to assess the relevance of the featured GPCR receptor for the model we knocked out the corresponding pathway by elimination of the respective ODE equations from our model and addressed the data fitting task with the reduced model. The result in the data space is shown in Figure 4C and indicates that the consideration of the GPCR feedback pathway in the model is in fact vital for reproducing the experimentally observed dynamics. For comparison we finally run the data fitting procedure with the parsimonious model (1) - (4) from [30]. The model lacks a GPCR mediated feedback pathway for fast response and fails to reproduce the corresponding experimental observation, see Figure 4D.

### Exploration of the dynamic repertoire of the model

**Figure 5:**
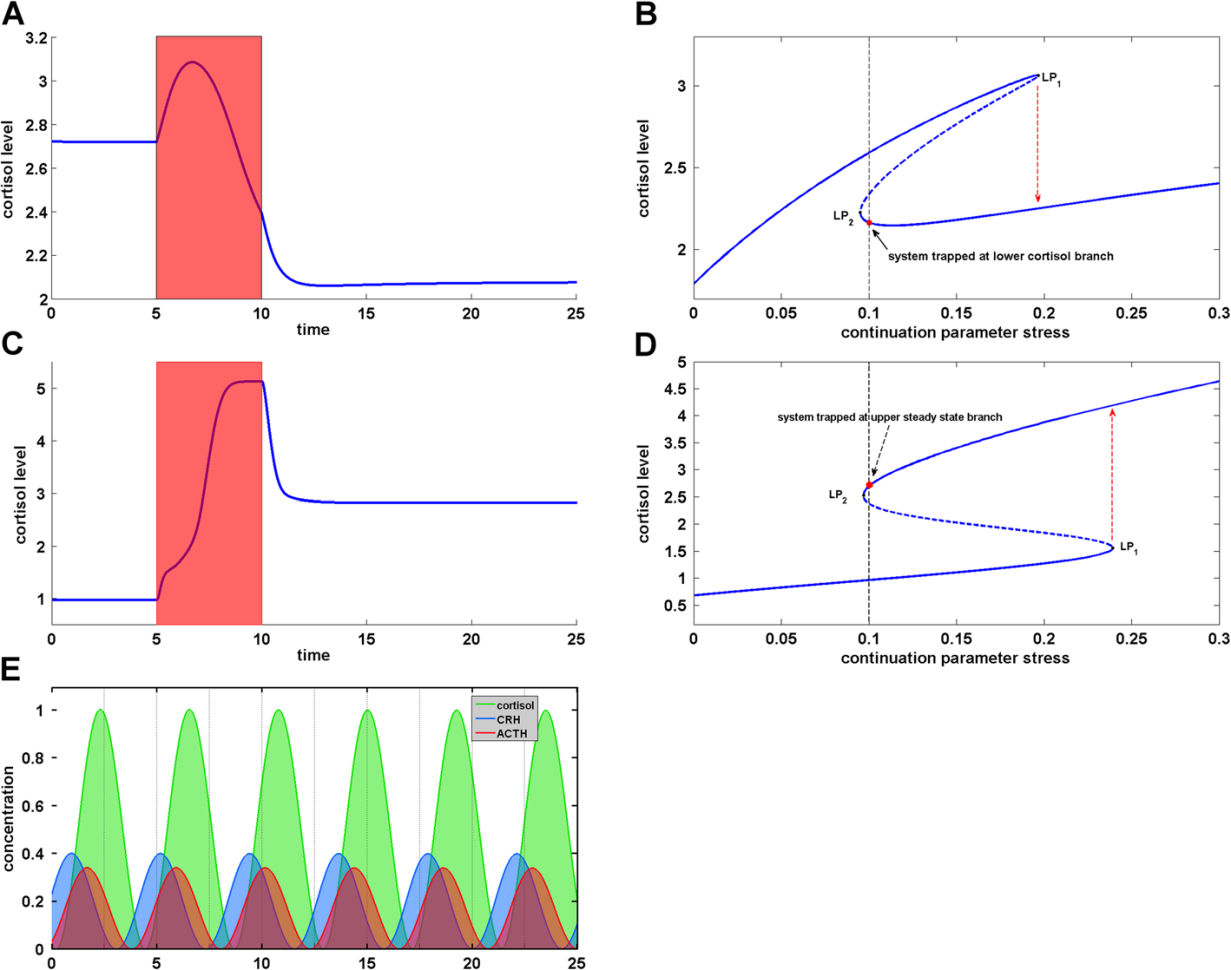
**Dynamic Repertoire of the HPA network model.** The figure displays different types of qualitative dynamic behavior featured by the HPA axis model from Figure 2 which is larger than for other HPA network models reported earlier in the literature. The corresponding model parameter settings have been found using inverse problems and bifurcation discovery tools. The bifurcation diagrams **(B)** and **(D)** show the steady state concentration of cortisol in dependence on the stress level. For a certain stress region the system exhibits two stable steady states (indicated by solid lines) and one unstable steady state (dashed line). **A)** Simulation of a dysfunctional stress response of cortisol in hypocortisolism. Though the stress is only temporary (red period), the system is driven to and eventually caught in an alternative stable steady state characterized by an insufficient cortisol level. **B)** Irreversible bistable switch explaining the behavior shown in **(A)**. Once the system is driven from the upper steady state branch it is trapped at the lower branch due to a limit point *LP*_2_ abscissa smaller than the basal stress level 0.1. **C)** Simulation of a dysfunctional stress response of cortisol in hypercortisolism. Though the stress is only temporary (red period), the system is driven to and eventually caught in an alternative stable steady state characterized by an excessive cortisol level. **D)** Irreversible bistable switch explaining the behavior shown in **(C)**. Note the inverted alignment of cortisol steady state branches and alternative location of limit points in comparison to **(B)**. **E)** Simulation of limit cycle oscillations in the hormone concentrations of CRH, ACTH and cortisol (relative to the maximum cortisol level).

One important aspect of [30] is the inclusion of the glucocorticoid receptor GR which generates bistability for a model based explanation of hypocortisolism as an irreversible biological switch. As the GR mediated feedback pathway is also a constituent of the extended HPA network model of Figure 2, the capability of our model to reflect the bistable behavior of (1)-(4) is an imperative requirement. Tools [46,47] for studying the dynamic repertoire of ODE systems with respect to bistability or oscillations solve optimization problems that involve the eigenvalues of the Jacobian matrix *f*_*x*_(*x*,*q*) (with *f* denoting the right hand side of an ODE model) and search for regions in the parameter space that give rise to limit-point or Hopf bifurcations. Figure 5B displays a resulting bifurcation diagram for the extended HPA model from Figure 2 with the stress level of Equation (8) as the bifurcation parameter. It gives an explanation of the dysfunctional alteration from a steady state of normal cortisol level (upper branch of the equilibrium curve) to a second steady state with low cortisol level (lower branch of the equilibrium curve) in response to a temporary stressor. If the abscissa of the limit point *LP*_2_ lies below the basal stress level, say for instance 0.1, the return to the upper branch is hampered and the HPA axis is trapped at low cortisol as observed in hypocortisolism. Figure 4A shows a corresponding cortisol concentration profile obtained by simulation of the model from Figure 2.

Further explorations of the dynamic repertoire of the extended HPA model revealed a region in the parameter space which allows for an alternative bistable behavior that can be linked to the pathological condition of hypercortisolism. Figure 5D provides an example of a corresponding bifurcation diagram in which the qualitative scenario of Figure 5B is inverted. If a stressor drives the HPA axis from the lower cortisol branch to the upper one, it abides at elevated cortisol levels even after removal of the stressor if the abscissa of the limit point *LP*_2_ lies below the basal stress level, again say for instance 0.1. Figure 5C shows a corresponding cortisol concentration profile obtained by simulation of the model depicted in Figure 2. While the positive feedback mediated by the glucocorticoid receptor *GR* dimerization has been already suggested in [30] as the cause for the bistable behavior associated to hypocortisolism, our in-silico pathway knock out tests on the extended HPA model indicate that the positive feedback on the genomic level mediated by the CRH hormone is vital for the bistability associated to hypercortisolism. Without this component, we could not find a parameter region giving rise to a bifurcation diagram as displayed in Figure 5D. Similarly, the results of our numerical analysis suggest that the dynamic repertoire of the parsimonious model of [30] excludes the bifurcation behavior of Figure 5D, possibly due to its simplistic description of the CRH pathway.

Finally, we explored the parameter space of the extended HPA network model with objective functions for the detection of Hopf bifurcations and found parameter regions allowing for stable limit cycle oscillations, see Figure 5E. The displayed temporal sequence in which the core species CRH, ACTH and cortisol reach their maximum value is in accordance with the established cascade in release of the HPA hormones. While oscillatory behavior for the parsimonious model was obtained in [30] in response to repeated stress pulses, limit cycle oscillation for (1)-(4) were found in [19] and for a modified version also in [32]. However, to our knowledge the HPA model shown in Figure 2 is the first one able to capture two distinct types of bistability (as reflected in Figures 5B and 5D) as well as autonomous oscillatory behavior.

### Model based correction of Hypercortisolism

Based on the parsimonious model of Figure 1 from [30] a therapeutic strategy for the correction of hypocortisolism has been suggested in [31]. This therapy aims at perturbing the HPA axis trapped at the low cortisol branch by a further suppression of cortisol in order to enable the return to a healthy cortisol level. An alternative idea for the treatment of hypocortisolism has been presented in [33] which aims at an intervention with the reaction network in order to turn the dysfunctional irreversible switch into a reversible one by shifting the critical limit point in the bifurcation diagram. As the extended HPA model of Figure 2 also captures a switching scenario associated to hypercortisolism, the inverse bifurcation approach of [33,48,48,49] can also be applied to study which parts of the HPA network need to be targeted in order to allow the return from the abnormal hypercortisol steady state back to a healthy cortisol level.

For the purpose of illustration we suppose that a representative *q*^0^ out of the parameter region compatible with the switching behavior of Figure 5D has been chosen (for instance by fitting of corresponding patient data) in which the abscissa *S*_2_(*q*^0^) of the limit point *LP*_2_(*q*^0^) lies below the basal stress level *S* = 0.1 (on a fictive scale). Then, a short but strong stress signal may drive the system from a normal cortisol level to an abnormal elevated one where it abides even if the stress drops back to basal level, see Figure 5C. A therapeutic qualitative change from an irreversible to a reversible steady state transition can be achieved by interfering with the HPA model such that the abscissa of the limit point *LP*_2_(*q*^0^) is shifted to a value $S_{2}^{*}$ with $S_{2}^{*}>0.1$ while, e.g., maintaining the abscissa value of the second limit point *LP*_1_(*q*^0^), see Figure 5A.

A corresponding interference strategy can be derived by solving the nonlinear inverse problem

(5) $$F(x)=\left(\begin{array}{c} S_{1}(q^{0})\\ S_{2}^{*} \end{array}\right),$$

where *F* maps the correction *x* onto the abscissas *S*_1_(*q*^0^ + *x*) and *S*_2_(*q*^0^ + *x*) of the limit points *LP*_1_(*q*^0^ + *x*) and *LP*_2_(*q*^0^ + *x*) defined by the HPA model with altered parameter vector *q*^0^ + *x*. In order to keep the number of necessary interventions with the HPA network as low as possible a sparse solution of (5) is sought for by solving the optimization problem

(6) $$\underset{q}{\text{min}}∥\left(\begin{array}{c} S_{1}(q^{0})\\ S_{2}^{*} \end{array}\right)-F(q){∥}_{2}^{2}+\alpha \varphi (x)$$

with *φ* as in (35) and the sparsity promoting choice *p* < 1.

**Figure 6:**
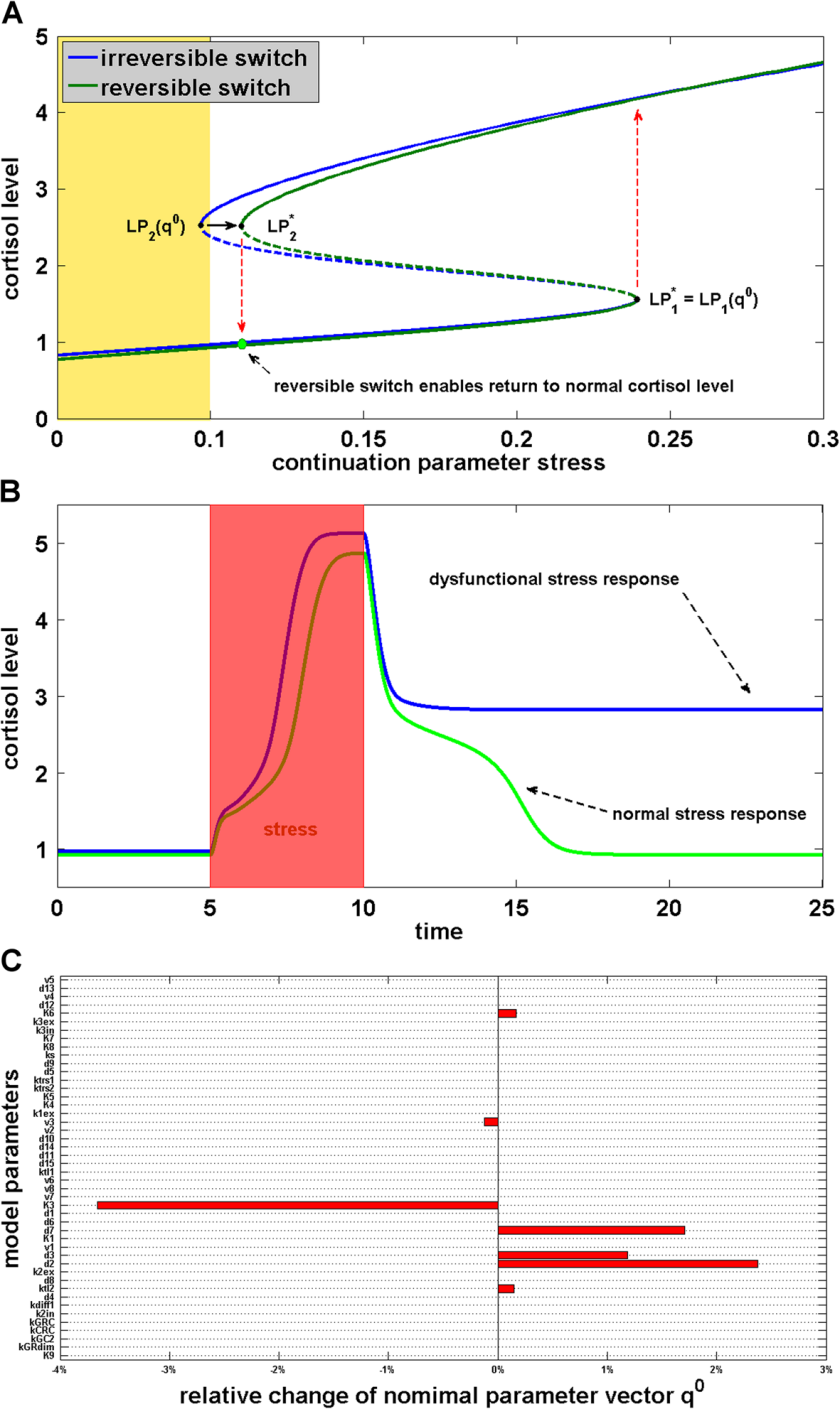
**Targeting the irreversible switch of the HPA axis model for hypercortisolism.** **A)** The blue equilibrium curve corresponds to the irreversible switch of hypercortisolism depicted also in Figure 4B. A shift of the limit point *LP*_2_(*q*^0^) to the region right to the basal stress level turns the irreversible switch into a reversible one (green equilibrium curve). This design goal can be formulated and solved using inverse problems techniques. **B)** Comparison of the dysfunctional stress response with the normal stress response after network manipulation allowing the return to normal cortisol levels following a short stress period (red area). **C)** Display of the necessary model parameter corrections *x* (relative to the nominal values of *q*^0^) in order to achieve the transition from an irreversible to a reversible switch as shown in Figure 5A. Due to the use of a sparsity promiting penalty function only a few parameters need to be altered.

Figure 6C displays the solution of (6) (in relation to *q*^0^) giving rise to the re-engineered bifurcation diagram of Figure 6A (green curve) and allowing the return to normal cortisol levels, see Figure 6B. The parameters *K*_6_, *v*_3_ and *d*_7_ to be altered concern the feedback controls related to the hormone CRH. A decrease of *v*_3_ and an increase of *d*_7_ correspond to a reduction of the sensitivity of the cells with respect to extracellular CRH. Moreover, an increase of the dissociation/Michaelis-Menten constant *K*_6_ reduces the sensitivity of the TFs transcription factor regulation by the CRH-CRHR related pathways. Furthermore, the increase of *k*_tl2_ calls for an increase of the translation rate of GR mRNA in order to further diminish the effect of extracellular CRH due to higher availability of GR. The intervention strategy shown in Figure 6C also suggests to alter the parameters *K*_3_, *d*_3_ and *d*_2_ in order to reduce the production of CRH and to increase the sensitivity with respect to the feedback via cortisol. As these parameters concern processes outside of the anterior pituitary gland cells, an extension of HPA model by a mechanistic description of the hypothalamus is necessary in order to enable a more detailed interpretation of those parameter changes.

## Conclusions

Motivated by the experimental detection of a glucocorticoid membrane receptor GPCR we have built a mathematical model of the anterior pituitary cell that differentiates between genomic and GPCR mediated non-genomic signaling pathways. After the integration of our model into a literature framework of the hypothalamus pituitary adrenal axis and the training against stress induced ACTH concentration profiles of murine AtT-20 pituitary tumor cells we obtained a dynamic repertoire capturing two types of bistable switches as well as stable limit cycle oscillations. While the first bistable switch has been previously associated to the diseased state of hypocortisolism and utilized in the development of therapeutic strategies, to our knowledge the second bistable switch is novel in the context of HPA axis modelling. Along similar lines it can be linked to another dysfunctionality of the HPA axis referred to as hypercortisolism in which a continued abidance of the axis at elevated cortisol levels is observed even after complete removal of the stressing factor. Our in-silico knockout studies in particular indicate that the positive feedback on the genomic level mediated by the CRH hormone is responsible for the generation of the hypercortisolism switch. Using inverse bifurcation analysis with sparsity enforcing penalization we finally detected key model parameters that need to be altered in order to turn the irreversible switch into a reversible one then featuring a healthy stress response characterized by a return to normal cortisol levels.

While systems biology strongly advertises the use of modeling and simulation in the design of future drugs and therapies, one current challenge is to appropriately picture the complexity of the biological system at hand in the defining mathematical equations and their parameters. Though with respect to HPA axis modeling we reached a certain level of detail by introducing both genomic and non-genomic signaling pathways in the pituitary cell, we only adopted the parsimonious description of the link between the pituitary and the hypothalamus as well as the adrenal from the literature. Furthermore, the information content of the available ACTH concentration did not allow to sufficiently constrain the model parameters such that we so far have to content ourselves with parameter space regions corresponding to different qualitative model behaviour. Nevertheless, even at its current stage our model offers to endocronologists a different view on hypercortisolism as an irreversible bistable switch while the outlined mathematical methodologies will also apply to more realistic models and improved data sets. Finally, the model may also serve as a tool for the design of a model predictive control based treatment of hypercortisolism in which the HPA axis is driven by a - though at first glance counter-intuitive - *additional administration* of cortisol to a point where the only stable condition in proximity is the original healthy state, compare with [31].

## Methods

### Model development

In order to distinguish between slow genomic and fast non-genomic processes we use the modeling concept of compartments and separate the nucleus from the cytoplasm of the pituitary gland cell. Moreover, we base our model on the principles of Michaelis-Menten enzyme kinetics ( [50-52]) in order to deal with physical meaningful parameters.

We refrain from a detailed description of gene regulation, RNA processing or cleaving and rather concentrate on the rate limiting processes such as transport/diffusion in and out of the nucleus. We apply this modeling strategy not only to the glucocorticoid mediated feedbacks but also to the stimulating pathways involving the hypothalamic hormone CRH. CRH does not enter the pituitary gland cells but interacts with a specific membrane receptor (CRHR) only. Still, also the binding of CRH induces both a genomic and a non-genomic feedback (cf. [53-55]). As this positive feedback via CRH counteracts the negative feedback via cortisol on the genomic as well as on the non-genomic level, also the catalyzing mechanisms related to CRH have to be modeled in more detail than in [30] for the purpose of differentiating between genomic and non-genomic effects in the anterior pituitary gland cells. Concerning the molecular mechanisms of the anterior pituitary cells, our model involves three different pathways which are associated with the three main receptors, namely the intracellular glucocorticoid receptor GR and the two membrane receptors (GPCR and CRHR) with respect to cortisol and CRH, see Figure 2 and Figure 3.

#### Extracellular species and the controls related to the adrenals and the hypothalamus

Regarding the feedback controls related to the adrenals and the hypothalamus we follow the approach of [30] in order to insure consistency and comparability as far as possible. In particular, we consider the following ODE describing the extracellular concentration of cortisol, with an additional term reflecting the diffusion (diffusion constant *k*_diff1_) of cortisol into the anterior pituitary cells:

(7) $$\frac{d[{\text{COR}}_{\text{ex}}]}{dt}=\frac{V_{\text{in}}}{V_{\text{ex}}}\;k_{\text{diff1}}([{\text{COR}}_{\text{in}}]-[{\text{COR}}_{\text{ex}}])+\frac{v_{1}[{\text{ACTH}}_{\text{ex}}]}{K_{1}+[{\text{ACTH}}_{\text{ex}}]}-d_{1}[{\text{COR}}_{\text{ex}}].$$

Here *V*_ex_ and *V*_in_ are the volumes of the extracellular compartment and the intracellular compartment. Analogously to [30] we model the temporal development of the CRH concentration by

(8) $$\frac{d[\text{CRH}]}{dt}=\frac{k_{s}\;\left(k_{\text{sb}}+\text{stress}\right)}{1+[{\text{COR}}_{\text{ex}}]/K_{3}}-d_{2}[\text{CRH}].$$

#### The GR mediated feedback control

One significant extension over [30] in our modeling approach concerns the regulatory pathway related to the intracellular glucocorticoid receptor GR and in particular to the transcription factor, being the homodimer of the GR-COR complex, see Figure 3A. The following equation describes the intracellular concentration of cortisol

(9) $$\begin{array}{ll} \;\frac{d[{\text{COR}}_{\text{in}}]}{dt} & =k_{\text{diff1}}\;([{\text{COR}}_{\text{ex}}]-[{\text{COR}}_{\text{in}}])-d_{4}[{\text{COR}}_{\text{in}}]\\ & \;+F\left(\left[{\left(\text{GR-COR}\right)}_{\text{2,nu}}],[{\text{COR}}_{\text{in}}],[\text{GR}\right]\right)\;. \end{array}$$

The change in the amount of cortisol in the cytoplasm (COR_in_) is subject to the transport of the dimerized GR-COR complex into the nucleus. This is described by

(10) $$\begin{array}{ll} \;F\left(\;,\left[{\left(\text{GR-COR}\right)}_{\text{2,nu}}],[{\text{COR}}_{\text{in}}],[\text{GR}\right],\;\right) & :=\bar{k}\;\left(\frac{k_{2\text{ex}}\;k_{\text{GRC}}^{2}\;k_{\text{GRdim}}[{\left(\text{GR-COR}\right)}_{\text{2,nu}}]}{{\left[{\text{COR}}_{\text{in}}\right]}^{2}\cdot [\text{GR}]+[{\text{COR}}_{\text{in}}]\cdot {\left[\text{GR}\right]}^{2}}\right)\\ & \;-\left(\frac{k_{2\text{in}}[{\text{COR}}_{\text{in}}]\cdot [\text{GR}]}{\left([{\text{COR}}_{\text{in}}]+[\text{GR}]\right)}\right)\;, \end{array}$$

which follows from the assumption of the fast binding of cortisol and GR and the quick formation of the homodimer. A detailed derivation is provided in the Additional file 1.

The dimerized GR-COR complex is transported to the nucleus, where it acts as a transcription factor and binds to the respective DNA sites. In particular it binds in the promoter region of the GR gene and enhances the transcription of the GR RNA sequences

(11) $$\frac{d[\text{pmGR}]}{dt}=-k_{\text{trs2}}[\text{pmGR}]+v_{7}+\frac{v_{8}[{\left(\text{GR-COR}\right)}_{\text{2,nu}}]}{K_{9}+[{\left(\text{GR-COR}\right)}_{\text{2,nu}}]}-d_{15}[\text{pmGR}]\;,$$

which then are transported to the cytoplasm and translated to the GR protein.

The second gene regulated by the GR-COR complex is the Proopiomelanocortin gene POMC, which yields the ACTH peptide after post-translational cleavage. While the POMC gene is up-regulated by certain transcription factors (TFs) related to the CRH stimulus, it is down-regulated by the GR-COR complex. It has been observed that these two transcription factors counteract via competitive inhibition (cf. [53,56]), i.e. the binding of the GR-COR complex prevents the catalyzation of the POMC expression and thus down regulates the transcription. This can be described by the following equation for the transcribed RNA

(12) $$\begin{array}{ll} \;\frac{d[\text{pmPOMC}]}{dt} & =v_{5}+\frac{v_{6}[{\text{TFs}}_{\text{nu}}]}{K_{7}\;\left(1+[{\left(\text{GR-COR}\right)}_{\text{2,nu}}]/K_{8}\right)+[{\text{TFs}}_{\text{nu}}]}\\ & \;-k_{\text{trs1}}[\text{pmPOMC}]-d_{14}[\text{pmPOMC}]\;. \end{array}$$

#### The GPCR mediated feedback control

The second pathway we consider in our model is the negative feedback on the release of ACTH via the glucocorticoid membrane receptor (GPCR), see Figure 3B. Concerning the production of the GPCR receptor we assume no regulation by means of other compounds and hence disregard the processes on the genomic level. With respect to the complex formation (COR-GPCR) we suppose a (quasi) equilibrium as it has been experimentally observed that two ligands bind to the receptor and exhibit positive cooperativity (cf. [43]).

The GPCR mediated feedback control only regulates the release of ACTH from intracellular vesicles. Again this mechanism counteracts the positive stimulus by CRH which is also detected via a membrane bound receptor (CRHR). It is well known that the anterior pituitary gland cells show a fast response to exposure with cortisol and CRH and that this is achieved by signaling cascades, directly relating the stimuli to the fusing of the vesicles (c.f. [36,37]). Consequently, we assume that the release of ACTH directly depends on the amount of formed receptor ligand complexes, where the catalysis of the CRH-receptor complex and the inhibitory effect of the cortisol-receptor complex are modeled as competitive inhibition. The following ODE describes the extracellular ACTH concentration

(13) $$\begin{array}{l} \frac{d[{\text{ACTH}}_{\text{ex}}]}{dt}=\frac{V_{\text{in}}}{V_{\text{ex}}}\;\frac{k_{1\text{ex}}[\text{CRHR-CRH}]\cdot [{\text{ACTH}}_{\text{in}}]}{K_{4}\;\left(1+[\text{GPCR-}{\text{(COR)}}_{2}]/K_{5}\right)+\text{CRHR-CRH}]}-d_{3}[{\text{ACTH}}_{\text{ex}}]\;. \end{array}$$

#### The CRHR mediated feedback control

The last pathway we consider concerns the feedback mechanism related to CRH and the respective membrane receptor CRHR, see Figure 3C. The main effects are the stimulated release of ACTH and the enhanced expression of the POMC gene. The genomic effects of the CRH mediated feedback involve several transcription factors (the Nur factors, Tpit/Tbx19, Pitx1, cf. [53]), which catalyze the expression of the POMC gene. However, for keeping the complexity moderate we only incorporate a single CRH related transcription pathway into our model and denote the representative factor by TFs, see Equation (12). The alternative would be to model each of the transcription pathways separately at the cost of additional equations and parameters to be identified, for details on transcription modeling at different levels see, e.g., [57]. Consequently, we also model the catalyzed production of TFs only in a coarse manner, where we account for the fast signaling of the binding of CRH onto the production of TFs by a hill factor, leading to

(14) $$\frac{d[{\text{TFs}}_{\text{in}}]}{dt}=\frac{V_{\text{nu}}}{V_{\text{in}}}\;k_{3\text{ex}}[{\text{TFs}}_{\text{nu}}]-k_{3\text{in}}[{\text{TFs}}_{\text{in}}]+\frac{v_{4}{\left[\text{CRHR-CRH}\right]}^{h}}{K_{6}^{h}+{\left[\text{CRHR-CRH}\right]}^{h}}-d_{12}[{\text{TFs}}_{\text{in}}]\;.$$

Concerning the fast response to CRH stimulation it is the common hypothesis that the release of ACTH is regulated via a signaling cascade which is induced by the binding of CRH to the CRHR receptor in the cell membrane. Signaling cascades are very fast compared to other cellular processes. In fact the response to cortisol or CRH has been observed already after a few micro seconds (cf. [40,54,58,59]). Thus we assume an immediate effect of the membrane receptor complex formation on the release of ACTH, as modeled in the ODE (13).

Figure 2 gives a graphical outline of the complete model which is mathematically described by means of the following non-linear ODE system with 46 modeling parameters in 15 equations

(15) $$\begin{array}{l} \;\frac{d[{\text{COR}}_{\text{ex}}]}{dt}=\frac{V_{\text{in}}}{V_{\text{ex}}}\;k_{\text{diff1}}([{\text{COR}}_{\text{in}}]-[{\text{COR}}_{\text{ex}}])+\frac{v_{1}[{\text{ACTH}}_{\text{ex}}]}{K_{1}+[{\text{ACTH}}_{\text{ex}}]}-d_{1}[{\text{COR}}_{\text{ex}}] \end{array}$$

(16) $$\begin{array}{l} \frac{d[\text{CRH}]}{dt}=\frac{k_{s}\;\left(k_{\text{sb}}+\text{stress}\right)}{1+[{\text{COR}}_{\text{ex}}]/K_{3}}-d_{2}[\text{CRH}] \end{array}$$

(17) $$\begin{array}{ll} \;\frac{d[{\text{ACTH}}_{\text{ex}}]}{dt} & =\frac{V_{\text{in}}}{V_{\text{ex}}}\;\frac{k_{1\text{ex}}[\text{CRHR-CRH}]\cdot [{\text{ACTH}}_{\text{in}}]}{K_{4}\;\left(1+[\text{GPCR-}{\text{(COR)}}_{2}]/K_{5}\right)+[\text{CRHR-CRH}]}\\ & \;-d_{3}[{\text{ACTH}}_{\text{ex}}] \end{array}$$

(18) $$\begin{array}{ll} \;\frac{d[{\text{COR}}_{\text{in}}]}{dt} & =k_{\text{diff1}}([{\text{COR}}_{\text{ex}}]-[{\text{COR}}_{\text{in}}])-d_{4}[{\text{COR}}_{\text{in}}]\\ & \;+F\left(\left[{\left(\text{GR-COR}\right)}_{\text{2,nu}}],[{\text{COR}}_{\text{in}}],[\text{GR}\right]\right) \end{array}$$

(19) $$\begin{array}{ll} \;\frac{d[{\text{ACTH}}_{\text{in}}]}{dt} & =k_{\text{tl1}}[\text{mPOMC}]-d_{5}[{\text{ACTH}}_{\text{in}}]\\ & \;-\frac{k_{1\text{ex}}[\text{CRHR-CRH}]\cdot [{\text{ACTH}}_{\text{in}}]}{K_{4}\;\left(1+[\text{GPCR-}{\text{(COR)}}_{2}]/K_{5}\right)+[\text{CRHR-CRH}]} \end{array}$$

(20) $$\begin{array}{l} \frac{d[\text{GPCR}]}{dt}=v_{2}-d_{6}[\text{GPCR}] \end{array}$$

(21) $$\begin{array}{l} \frac{d[\text{CRHR}]}{dt}=v_{3}-d_{7}[\text{CRHR}] \end{array}$$

(22) $$\begin{array}{l} \frac{d[\text{GR}]}{dt}=k_{\text{tl2}}[\text{mGR}]+F\left(\left[{\left(\text{GR-COR}\right)}_{\text{2,nu}}],[{\text{COR}}_{\text{in}}],[\text{GR}\right]\right)-d_{8}[\text{GR}] \end{array}$$

(23) $$\begin{array}{l} \frac{d[{\left(\text{GR-COR}\right)}_{\text{2,nu}}]}{dt}=k_{2\text{in}}[{\left(\text{GR-COR}\right)}_{\text{2,in}}]-k_{2\text{ex}}[{\left(\text{GR-COR}\right)}_{\text{2,nu}}]-d_{9}[{\left(\text{GR-COR}\right)}_{\text{2,nu}}] \end{array}$$

(24) $$\begin{array}{l} \;\frac{d[\text{mPOMC}]}{dt}=\frac{V_{\text{nu}}}{V_{\text{in}}}\;k_{\text{trs1}}[\text{pmPOMC}]-d_{10}[\text{mPOMC}] \end{array}$$

(25) $$\begin{array}{l} \frac{d[\text{mGR}]}{dt}=\frac{V_{\text{nu}}}{V_{\text{in}}}\;k_{\text{trs2}}[\text{pmGR}]-d_{11}[\text{mGR}] \end{array}$$

(26) $$\begin{array}{ll} \;\frac{d[{\text{TFs}}_{\text{in}}]}{dt} & =\frac{V_{\text{nu}}}{V_{\text{in}}}\;k_{3\text{ex}}[{\text{TFs}}_{\text{nu}}]-k_{3\text{in}}[{\text{TFs}}_{\text{in}}]-d_{12}[{\text{TFs}}_{\text{in}}]\\ & \;+\frac{v_{4}\;{\left[\text{CRHR-CRH}\right]}^{h}}{K_{6}^{h}+{\left[\text{CRHR-CRH}\right]}^{h}} \end{array}$$

(27) $$\begin{array}{l} \frac{d[{\text{TFs}}_{\text{nu}}]}{dt}=k_{3\text{in}}[{\text{TFs}}_{\text{in}}]-k_{3\text{ex}}[{\text{TFs}}_{\text{nu}}]-d_{13}[{\text{TFs}}_{\text{nu}}] \end{array}$$

(28) $$\begin{array}{ll} \;\frac{d[\text{pmPOMC}]}{dt} & =-k_{\text{trs1}}[\text{pmPOMC}]+v_{5}-d_{14}[\text{pmPOMC}]\\ & \;+\frac{v_{6}[{\text{TFs}}_{\text{nu}}]}{K_{7}\;\left(1+[{\left(\text{GR-COR}\right)}_{\text{2,nu}}]/K_{8}\right)+[{\text{TFs}}_{\text{nu}}]} \end{array}$$

(29) $$\begin{array}{l} \frac{d[\text{pmGR}]}{dt}=-k_{\text{trs2}}[\text{pmGR}]+v_{7}+\frac{v_{8}[{\left(\text{GR-COR}\right)}_{\text{2,nu}}]}{K_{9}+[{\left(\text{GR-COR}\right)}_{\text{2,nu}}]}-d_{15}[\text{pmGR}], \end{array}$$

where

(30) $$\begin{array}{ll} \;F\left(\;[{\left(\text{GR-COR}\right)}_{\text{2,nu}}],[{\text{COR}}_{\text{in}}],[\text{GR}]\;\right) & :=\bar{k}\;\left(\frac{k_{2\text{ex}}\;k_{\text{GRC}}^{2}\;k_{\text{GRdim}}[{\left(\text{GR-COR}\right)}_{\text{2,nu}}]}{{\left[{\text{COR}}_{\text{in}}\right]}^{2}\cdot [\text{GR}]+[{\text{COR}}_{\text{in}}]\cdot {\left[\text{GR}\right]}^{2}}\right)\\ & \;-\left(\frac{k_{2\text{in}}[{\text{COR}}_{\text{in}}]\cdot [\text{GR}]}{\left([{\text{COR}}_{\text{in}}]+[\text{GR}]\right)}\right) \end{array}$$

with $\bar{k}=\frac{V_{\text{nu}}}{2\;V_{\text{in}}}$.

#### Model parameters

We now comment on the role of the parameters in our ODE model. We have three subsidiary parameters describing the average volumes of the three compartments *V*_in_, *V*_ex_ and *V*_nu_. These parameters are phenomenological and are fixed in the subsequent analysis.

Another class of parameters are the constants related to diffusion and transport of the compounds. In our model *k*_diff1_, *k*_1ex_, *k*_2in_, *k*_2ex_, *k*_3ex_ and *k*_3in_ comprise the list of these parameters. We do not explicitly model active transport, as this would involve additional regulatory species and mechanisms and consequently would lead to an even more complex model. Subsequently, we assume that the rate of diffusion/transport is constant on average.

The largest portion of model parameters is related to degradation processes whose rates are assumed to be proportional to the concentration of the respective species. The associated rate constants are denoted by *d*_1_, *d*_2_, …, *d*_14_ and *d*_15_.

As a consequence of the considered enzymatic reactions and ligand-receptor complexes the model includes several dissociation constants. Moreover, we have some constants arising due to the Michaelis-Menten approximations, i.e. the maximum reaction rates and the Michaelis-Menten constants. They are closely related to the dissociation constant of the respective complex and approximate them if the rate of the product formation dominates the degradation of the enzyme-substrate complex. *k*_GRdim_, *k*_GC2_, *k*_CRC_ and *k*_GRC_ denote the dissociation constants of the considered ligand-receptor complexes. *K*_1_, *K*_3_, …, *K*_9_ denote the Michaelis-Menten constants of the respective enzyme-substrate complexes. *v*_1_, *k*_s_, *k*_s_*k*_sb_ and *v*_4_ denote the corresponding limiting rates of the Michaelis-Menten approximations.

Our description of the genomic mechanisms involves rates for transcription (*v*_5_, *v*_6_, *v*_7_ and *v*_8_), translation (*k*_tl1_ and *k*_tl2_) and transport (*k*_trs1_ and *k*_trs2_) of mRNA. We neglect the post-processing of mRNA as our focus is on the respective timescales and as the time delay is mainly due to the translocation. The constants *v*_2_ and *v*_3_ denote the production rate of the two membrane receptors. As we have not considered their regulatory mechanism we lumped the transcription and translation rate together.

### Model training against data

The central idea of variational regularization is to augment the least-squares term that measures the mismatch between the data and the model with a penalizing term *φ* that guarantees the continuous dependence of the parameter solution on the data

(31) $$\begin{array}{ll} & {∥x(q,\rho_{1})-y_{1}^{\delta }∥}_{2}^{2}+{∥x(q,\rho_{2})-y_{2}^{\delta }∥}_{2}^{2}+\alpha \varphi (q)\;\to \;\underset{\left(q,\rho_{1},\rho_{2}\right)}{\text{min}} \end{array}$$

(32) $$\begin{array}{l} \text{subject to:}\;\dot{x}(q,\rho_{k})=f(x,q) \end{array}$$

(33) $$\begin{array}{l} \;x(q,\rho_{k})=\rho_{k}\;k\in \{1,2\} \end{array}$$

(34) $$\begin{array}{l} \;c(\rho_{k})\leq 0\;k\in \{1,2\} \end{array}$$

**Figure 7:**
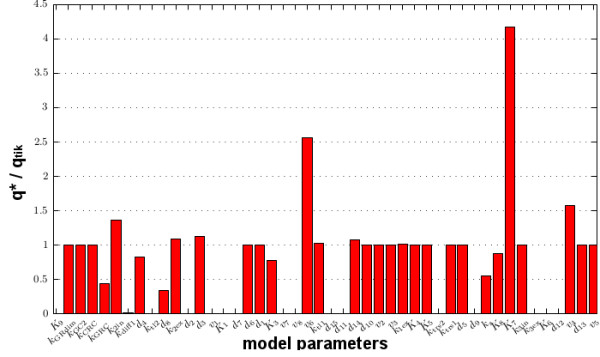
**Comparison of parameter solutions for data fitting.** Plot of *q*^∗^/*q*^*tik*^ (in componentwise sense) with the parameter vectors *q*^∗^ and *q*^*tik*^ as inferred from the experimental data of Figure 4A in a two step approach. The result shows that certain pathways of the model are of minor relevance for the reproduction of the experimentally observed dynamics as given in Figure 4B.

The ODE system (32) represents the HPA axis model of Figure 2 along with its parameter vector *q* of length 46. *ρ*_1_ and *ρ*_2_ in (33) denote the initial conditions corresponding to the two different experimental settings as described above and the two resulting data sets $y_{1}^{\delta }$ and $y_{2}^{\delta }$ displayed in Figure 5A. While the initial concentration of some species are known from the experimental setup and can be taken into account by the constraints (34), the remaining components of *ρ*_1_, *ρ*_2_ have to be estimated from the data as well. As the available amount of data is not sufficient to uniquely determine the model parameters, we have chosen a two step approach for finding the model components most relevant for reproducing the data. First, we applied classical Tikhonov regularization [45] and solved (31) with *p* = 2 in the penalty term

(35) $$\varphi (q)=\underset{i}{\sum }|q_{i}{|}^{p}$$

using a combination of global and local optimization routines. The resulting parameter vector *q*^*tik*^ then served as an initial guess for solving (31) with a *p*-value less than one. Sparsity enforcing *ℓ*_*p*_ functionals with the choice 0 < *p* ≤ 1 aim at solutions whose less important components are driven to zero [60-62] and have been used in the context of parameter identification in [63,64]. For nonlinear inverse problems, the regularization properties of (35) with 0 < *p* ≤ 1 have been analyzed in [65,66]. Figure 7 displays the parameter vector *q*^∗^ obtained for *p* = 0.9 in relation to *q*^*tik*^. The near zero components of *q*^∗^ correspond to reactions and pathways that can rather be neglected in fitting the experimental data, see Figure 5B. In particular, the autocatalytic loop for the GR gene regulation, represented by the parameters *k*_trs_, *v*_7_, *v*_8_, *v*_9_, *d*_15_, *d*_11_, seems negligible with respect to the fast dynamics. Furthermore, the near zero value of *K*_6_ indicates that the production of the TFs receptors is independent of CRH, i.e. the genomic part of the CRH related feedback.

### Origin of experimental data

Murine anterior pituitary AtT-20 cells (passage 12-24) were seeded and maintained as previously described [44]. After 92 hours of cell growth AtT-20 cells were exposed to doses of 10nM CRH and in addition 100nM cortisol. The supernatant was carefully removed from the cell layer after 1 minute to 1 hour of administration and centrifuged for 10 minutes with 800xg at 37°C. The concentration of ACTH-molecules in the cell-free supernatant was detected using Fluorescence Correlation Spectroscopy Immunoassay [44].

The AtT-20 cells (ATCC no. CCL-89) were purchased from the American Type Culture Collection (ATCC, Manassas, USA). Cell culture media and reagents were from Sigma (Sigma-Aldrich Inc., St. Louis, MI).

## Competing interests

The authors declare that they have no competing interests.

## Authors’ contributions

PK and CZ wrote the manuscript, discussed and simulated the HPA model, CZ developed the HPA model, MP conducted the lab experiments, MP and GK designed the lab experiments. All authors read and approved the final manuscript.

## Supplementary Material

Additional file 1 Detailed derivation of the mathematical model. Click here for file

## References

[B1] Alberts B Johnson A Lewis J Raff M Roberts K Walter P Molecular Biology of the Cell: Reference Edition 2007 Garland Science

[B2] Melmed S Kleinberg D Ho K Melmed S, Polonsky KS, Larsen PR, Kronenberg HM Pituitary Physiology and Diagnostic Evaluation Williams Textbook of Endocrinology: Expert Consult-Online and Print 2011 Philadelphia: Elsevier/Saunders 175 228

[B3] Habib KE Gold PW Chrousos GP Neuroendocrinology of stress Endocrinol Metab Clin North Am 2001 10 695 728 10.1016/S0889-8529(05)70208-5 11571937

[B4] Papadimitriou A Priftis KN Regulation of the hypothalamic-pituitary-adrenal axis Neuroimmunomodulation 2009 10 265 271 10.1159/000216184 19571587

[B5] Demitrack MA Dale JK Straus SE Laue L Listwak SJ Kruesi MJ Chrousos GP Gold PW Evidence for impaired activation of the hypothalamic-pituitary-adrenal axis in patients with chronic fatigue syndrome J Clin Endocrinol Metab 1991 10 1224 1234 10.1210/jcem-73-6-1224 1659582

[B6] Cleare AJ The HPA axis and the genesis of chronic fatigue syndrome Trends Endocrinol Metab 2004 10 55 59 10.1016/j.tem.2003.12.002 15036250

[B7] Di Giorgio A Hudson M Jerjes W Cleare AJ 24-hour pituitary and adrenal hormone profiles in chronic fatigue syndrome Psychosom Med 2005 10 433 440 10.1097/01.psy.0000161206.55324.8a 15911907

[B8] Jerjes WK Peters TJ Taylor NF Wood PJ Wessely S Cleare AJ Diurnal excretion of urinary cortisol, cortisone, and cortisol metabolites in chronic fatigue syndrome J Psychosom Res 2006 10 145 153 10.1016/j.jpsychores.2005.07.008 16439267

[B9] Yehuda R Advances in understanding neuroendocrine alterations in PTSD and their therapeutic implications Ann New York Acad Sci 2006 10 137 166 [ http://dx.doi.org/10.1196/annals.1364.012] 10.1196/annals.1364.012 16891568

[B10] Juruena MF Cleare AJ Pariante CM The hypothalamic pituitary adrenal axis, glucocorticoid receptor function and relevance to depression Rev Bras Psiquiatr 2004 10 189 201 10.1590/S1516-44462004000300009 15645065

[B11] Gold PW Chrousos GP Organization of the stress system and its dysregulation in melancholic and atypical depression: high vs low CRH/NE states Mol Psychiatry 2002 10 254 275 10.1038/sj.mp.4001032 11920153

[B12] Varghese FP Brown ES The Hypothalamic-Pituitary-Adrenal Axis in major depressive disorder: a brief primer for primary care physicians Prim Care Companion J Clin Psychiatry 2001 10 151 155 10.4088/PCC.v03n0401 15014598 PMC181180

[B13] Lupien SJ Nair NPV Briére S Maheu F Tu MT Lemay M McEwen BS Meaney MJ Increased cortisol levels and impaired cognition in human aging Implication for depression and dementia in later life Rev Neurosci 2011 10 2 91 173 10.1515/revneuro.1999.10.2.117 10658955

[B14] Plaschke K Kopitz J Mattern J Martin E Teschendorf P Increased cortisol levels and anticholinergic activity in cognitively unimpaired patients J Neuropsychiatry Clinic Neurosci 2010 10 4 433 441 10.1176/appi.neuropsych.22.4.433 21037129

[B15] Mu DL Wang DX Li LH Shan GJ Li J Yu QJ Shi CX High serum cortisol level is associated with increased risk of delirium after coronary artery bypass graft surgery: a prospective cohort study Critic Care 2010 10 6 R238 [ http://ccforum.com/content/14/6/R238]. [See related commentary by Kazmierski and Kloszewska, http://ccforum.com/content/15/1/102] 10.1186/cc9393 PMC3219980 21192800

[B16] Hood L Heath JR Phelps ME Lin B Systems biology and new technologies enable predictive and preventative medicine Science 2004 10 5696 640 643 [ http://www.sciencemag.org/content/306/5696/640.abstract] 10.1126/science.1104635 15499008

[B17] Butcher EC Berg EL Kunkel EJ Systems biology in drug discovery Nature Biotechnol 2004 10 1253 1259 10.1038/nbt1017 15470465

[B18] Kuepfer L Lippert J Eissing T Goryanin II, Goryachev AB Multiscale mechanistic modeling in pharmaceutical research and development Advances in Systems Biology, Volume 736 of Advances in Experimental Medicine and Biology 2012 New York: Springer 543 561 10.1007/978-1-4419-7210-1_32 22161351

[B19] Lu J August E Koeppl H Inverse problems from biomedicine J Math Biol 2012 1 26 [ http://dx.doi.org/10.1007/s00285-012-0523-z] 10.1007/s00285-012-0523-z 22526835

[B20] Nielsen K Pociot F Ottesen J Bifurcation analysis of an existing mathematical model reveals novel treatment strategies and suggests potential cure for type 1 diabetes Math Med Biol (Online) 2013 10.1093/imammb/dqt006 23620354

[B21] Li G Liu B Liu Y A dynamical model of the pulsatile secretion of the hypothalamo-pituitary-thyroid axis Biosystems 1995 10 83 92 10.1016/0303-2647(94)01484-O 7772725

[B22] Liu BZ Peng JH Sun YC Liu YW A comprehensive dynamical model of pulsatile secretion of the hypothalamo-pituitary-gonadal axis in man Comput Biol Med 1997 10 6 507 13 10.1016/S0010-4825(97)00026-7 9437552

[B23] Kyrylov V Severyanova LA Vieira A Modeling robust oscillatory behavior of the hypothalamic-pituitary-adrenal axis IEEE Trans Biomed Eng 2005 10 12 1977 1983 10.1109/TBME.2005.857671 16366221

[B24] Bairagi N Chatterjee S Chattopadhyay J Variability in the secretion of corticotropin-releasing hormone, adrenocorticotropic hormone and cortisol and understandability of the hypothalamic-pituitaryadrenal axis dynamics–a mathematical study based on clinical evidence Math Med Biol 2008 10 37 63 10.1093/imammb/dqn003 18343885

[B25] Lenbury Y Pornsawad P A delay-differential equation model of the feedback-controlled hypothalamus-pituitary-adrenal axis in humans Math Med Biol 2005 10 15 33 [ http://imammb.oxfordjournals.org/content/22/1/15.abstract] 10.1093/imammb/dqh020 15716298

[B26] Vinther F Andersen M Ottesen J The minimal model of the hypothalamic–pituitary–adrenal axis J Math Biol 2011 10 663 690 [ http://dx.doi.org/10.1007/s00285-010-0384-2] 10.1007/s00285-010-0384-2 21107577

[B27] Ottesen J Mathematical modelling of the Hypothalamic-Pituritary-Adrenal glad (HPA)-axis; Including Hippocampal mechanicsms Math Biosci 2013 10 122 138 10.1016/j.mbs.2013.08.010 24012602

[B28] Savic D Jelic S A mathematical model of the hypothalamo-pituitary-adrenocortical system and its stability analysis Chaos, Solitons & Fractals 2005 10 2 427 436 [ http://www.sciencedirect.com/science/article/pii/S0960077905000810] 10.1016/j.chaos.2005.01.013

[B29] Jelic S Cupic Z Kolar-Anic L Mathematical modeling of the hypothalamic-pituitary-adrenal system activity Math Biosci 2005 10 2 173 187 10.1016/j.mbs.2005.06.006 16112688

[B30] Gupta S Aslakson E Gurbaxani BM Vernon SD Inclusion of the glucocorticoid receptor in a hypothalamic pituitary adrenal axis model reveals bistability Theor Biol Med Model 2007 10 8 10.1186/1742-4682-4-8 17300722 PMC1804264

[B31] Ben-Zvi A Vernon SD Broderick G Model-based therapeutic correction of hypothalamic-pituitary-adrenal axis dysfunction PLoS Comput Biol 2009 10 e1000273 [ http://dx.doi.org/10.1371%2Fjournal.pcbi.1000273] 10.1371/journal.pcbi.1000273 19165314 PMC2613527

[B32] Sriram K Rodriguez-Fernandez M Doyle FJ III Modeling cortisol dynamics in the neuro-endocrine axis distinguishes normal, depression, and post-traumatic stress disorder (PTSD) in humans PLoS Comput Biol 2012 10 2 e1002379 10.1371/journal.pcbi.1002379 22359492 PMC3280965

[B33] Engl HW Flamm C Kügler P Lu J Müller S Schuster P Inverse problems in systems biology Inverse Problems 2009 10 12 123014 [ http://stacks.iop.org/0266-5611/25/i=12/a=123014] 10.1088/0266-5611/25/12/123014

[B34] Tsai SY Carlstedt-Duke J Weigel NL Dahlman K Gustafsson JA Tsai MJ O’Malley BW Molecular interactions of steroid hormone receptor with its enhancer element: evidence for receptor dimer formation Cell 1988 10 361 369 10.1016/0092-8674(88)90059-1 3167984

[B35] Drouin J Sun YL Tremblay S Lavender P Schmidt TJ de Lean A Nemer M Homodimer formation is rate-limiting for high affinity DNA binding by glucocorticoid receptor Mol Endocrinol 1992 10 1299 1309 10.1210/me.6.8.1299 1406707

[B36] Norman AW Mizwicki MT Norman DP Steroid-hormone rapid actions membrane receptors and a conformational ensemble model Nat Rev Drug Discov 2004 10 27 41 10.1038/nrd1283 14708019

[B37] Losel R Wehling M Nongenomic actions of steroid hormones Nat Rev Mol Cell Biol 2003 10 46 56 10.1038/nrm1009 12511868

[B38] Dayanithi G Antoni FA Rapid as well as delayed inhibitory effects of glucocorticoid hormones on pituitary adrenocorticotropic hormone release are mediated by type II glucocorticoid receptors and require newly synthesized messenger ribonucleic acid as well as protein Endocrinol 1989 10 308 313 10.1210/endo-125-1-308 2544406

[B39] Redei E Li L Halasz I McGivern RF Aird F Fast glucocorticoid feedback inhibition of ACTH secretion in the ovariectomized rat: effect of chronic estrogen and progesterone Neuroendocrinology 1994 10 113 23 [PMID: 7969768] 10.1159/000126741 7969768

[B40] Tasker JG Di S Malcher-Lopes R Minireview: rapid glucocorticoid signaling via membrane-associated receptors Endocrinology 2006 10 12 5549 56 10.1210/en.2006-0981 16946006 PMC3280589

[B41] Lightman SL Patterns of exposure to glucocorticoid receptor ligand Biochemical Society Transactions 2006 10 1117 8 [PMID: 17073764] 17073764 10.1042/BST0341117

[B42] Stellato C Post-transcriptional and nongenomic effects of glucocorticoids Proc Am Thorac Soc 2004 10 3 255 263 10.1513/pats.200402-015MS 16113443

[B43] Maier C Runzler D Schindelar J Grabner G Waldhausl W Kohler G Luger A G-protein-coupled glucocorticoid receptors on the pituitary cell membrane J Cell Sci 2005 10 Pt 15 3353 3361 16079279 10.1242/jcs.02462

[B44] Puchinger M Zarzer C Kügler P Gaubitzer E Kohler G In vitro detection of adrenocorticotropic hormone levels by fluorescence correlation spectroscopy immunoassay for mathematical modeling of glucocorticoid-mediated feedback mechanisms EURASIP J Bioinform Sys Biol 2012 10 17 [ http://bsb.eurasipjournals.com/content/2012/1/17] 10.1186/1687-4153-2012-17 PMC3502540 23102048

[B45] Engl HW Hanke M Neubauer A Regularization of Inverse Problems, Volume 375 of Mathematics and its Applications 1996 Dordrecht: Kluwer Academic Publishers Group

[B46] Chickarmane V Paladugu SR Bergmann F Sauro HM Bifurcation discovery tool Bioinformatics 2005 10 18 3688 3690 [ http://bioinformatics.oxfordjournals.org/content/21/18/3688.abstract] 10.1093/bioinformatics/bti603 16081475

[B47] Lu J Inverse eigenvalue problems for exploring the dynamics of systems biology models J Biol Eng 2009 10 711 728

[B48] Lu J Engl HW Schuster P Inverse bifurcation analysis: application to simple gene systems Algo Mol Biol 2006 10 11 3353 3361 10.1186/1748-7188-1-11 PMC1570349 16859561

[B49] Lu J Engl HW Machné R Schuster P Hochreiter S, Wagner R Inverse Bifurcation Analysis of a Model for the Mammalian G1/S Regulatory Module Bioinformatics Research and Development Volume 4414 of Lecture Notes in Computer Science 2007 Heidelberg: Springer Berlin 168 184 [ http://dx.doi.org/10.1007/978-3-540-71233-614]

[B50] Bisswanger H Enzyme Kinetics: Principles and Methods 2002 Weinheim: Wiley-VCH

[B51] Leskovac V Comprehensive Enzyme Kinetics 2004 New York, Boston, Dordrecht, London, Moscow: Kluwer Academic, Publishers

[B52] Cornish-Bowden A Fundamentals of Enzyme Kinetics 2004 Weinheim: Portland press

[B53] Lu L Suzuki T Yoshikawa Y Murakami O Miki Y Moriya T Bassett MH Rainey WE Hayashi Y Sasano H Nur-related factor 1 and nerve growth factor-induced clone B in human adrenal cortex and its disorders J Clin Endocrinol Metab 2004 10 4113 4118 10.1210/jc.2004-0069 15292355

[B54] Aguilera G Corticotropin releasing hormone, receptor regulation and the stress response Trends Endocrinol Metab 1998 10 8 329 336 10.1016/S1043-2760(98)00079-4 18406298

[B55] Ma XM Aguilera G Differential regulation of corticotropin-releasing hormone and vasopressin transcription by glucocorticoids Endocrinology 1999 10 12 5642 5650 10.1210/en.140.12.5642 10579328

[B56] Murakami I Takeuchi S Kudo T Sutou S Takahashi S Corticotropin-releasing hormone or dexamethasone regulates rat proopiomelanocortin transcription through Tpit/Pitx-responsive element in its promoter J Endocrinol 2007 10 2 279 290 10.1677/JOE-06-0143 17470519

[B57] Wilkinson DJ Stochastic Modelling for Systems Biology 2012 Boca Raton, London, New York: CRC Press

[B58] Hashimoto K Yunoki S Takahara J Ofuji T ACTH release in pituitary cell cultures. Effect of neurogenic peptides and neurotransmitter substances on ACTH release induced by hypothalamic corticotropin releasing factor (CRF) Endocrinol Jpn 1979 10 103 109 10.1507/endocrj1954.26.103 35343

[B59] Lowry PJ Estivariz FE Gillies GE Kruseman AC Linton EA CRF its regulation of ACTH and pro-opiomelanocortin peptide release and its extra hypothalamic occurrence Acta Endocrinol Suppl (Copenh) 1986 10 56 62 3019060 10.1530/acta.0.111s0056

[B60] Bruckstein AM Donoho DL Elad M From sparse solutions of systems of equations to sparse modeling of signals and images SIAM Rev 2009 10 11 34 81

[B61] Lai MJ On sparse solution of underdetermined linear systems J Concrete and Appl Math 2010 10 296 327

[B62] Daubechies I Defrise M De-Mol C An iterative thresholding algorithm for linear inverse problems with a sparsity constraint Commun on Pure Appl Math 2004 10 11 1413 1457 10.1002/cpa.20042

[B63] Bonneau R Reiss D Shannon P Facciotti M Hood L Baliga N Thorsson V The Inferelator: an algorithm for learning parsimonious regulatory networks from systems-biology data sets de novo Genome Biol 2006 10 5 R36 [ http://genomebiology.com/2006/7/5/R36] 10.1186/gb-2006-7-5-r36 16686963 PMC1779511

[B64] Kügler P Gaubitzer E Müller S Parameter identification for chemical reaction systems using sparsity enforcing regularization: a case study for the chlorite-iodide reaction J Phys Chemis A 2009 10 12 2775 2785 10.1021/jp808792u 19243161

[B65] Zarzer CA On Tikhonov regularization with non-convex sparsity constraints Inverse Problems 2009 10 025006 10.1088/0266-5611/25/2/025006

[B66] Grasmair M Haltmeier M Scherzer O Sparse regularization with *ℓ*_*q*_ penalty term Inverse Problems 2008 10 5 055020 [ http://stacks.iop.org/0266-5611/24/i=5/a=055020] 10.1088/0266-5611/24/5/055020

