# Supplementary material for: Differentiation between genomic and non-genomic feedback controls yields an HPA axis model featuring Hypercortisolism as an irreversible bistable switch
Source: Theor Biol Med Model. 2013 Nov 9;10:65. doi: 10.1186/1742-4682-10-65 (PMC3879227; doi:10.1186/1742-4682-10-65)
Supplement: Additional file 1 — Detailed derivation of the mathematical model. [file 1742-4682-10-65-S1.pdf]

Detailed Derivation of the Mathematical Model

We develop our model by separately considering the different pathways. We refer to [1] for a more elaborate and extensive discussion of the modeling. We start with the ACTH production, transport and release and the interactions of the adrenals and the Hypothalamus. Concerning the interactions between the glands we closely follow the approach in [2].

The first ODE concerns the extracellular concentration of cortisol, which is modulated by the exchange with the anterior pituitary cells (diffusion), the degradation of the molecule and the secretion from the adrenal cortex. Due to the focus on the anterior pituitary cells we simplify the secretion process from the adrenal cortex. For that purpose we assume a saturating response with respect to the stimulus of ACTH of the form:

$$\frac{v_1\,[\mathrm{ACTH_{ex}}]}{K_1 + [\mathrm{ACTH_{ex}}]}\,, \tag{1}$$

where v_1 is the limiting secretion rate of cortisol from the adrenal cortex and K_1 represents the sensitivity of the ACTH affine receptors in the adrenal cortex. Altogether, this leads to the following ODE for the extracellular cortisol concentration:

$$\frac{\mathrm{d}[\mathrm{COR_{ex}}]}{\mathrm{d}t} = \frac{V_{\mathrm{in}}}{V_{\mathrm{ex}}}\,k_{\mathrm{diff1}}\left([\mathrm{COR_{in}}] - [\mathrm{COR_{ex}}]\right) + \frac{v_1\,[\mathrm{ACTH_{ex}}]}{K_1 + [\mathrm{ACTH_{ex}}]} - d_1\,[\mathrm{COR_{ex}}]\,, \tag{2}$$

where V_{ex} and V_{in} are the volume sizes of the extracellular compartment and the intracellular compartment (including all cytoplasmic organelles except for the nucleus).

The next extracellular process we model is the secretion of CRH from the Hypothalamus. The release of CRH is regulated by the amount of stress signals (neurotransmitters) from the nervous system and the blood cortisol level. We model this by the following ODE:

$$\frac{\mathrm{d}[\mathrm{CRH}]}{\mathrm{d}t} = \frac{k_s\,\left(k_{\mathrm{sb}} + \mathrm{stress}\right)}{1 + [\mathrm{COR_{ex}}]/K_3} - d_2\,[\mathrm{CRH}]\,. \tag{3}$$

K_3 stands for the sensitivity of the cortisol affine receptors in the Hypothalamus. k_s represents the limiting secretion rate of CRH. k_{sb} describes the average basal secretion rate of CRH from the Hypothalamus, irrespective of the current stress level.

The first intracellular species we consider is $\mathrm{COR_{in}}$. The concentration is modified by the already considered flux trough the membrane of the pituitary gland cells and by the transport of the dimerized GR complex into the nucleus. This gives rise to a complex non-linear dependency. Since this is additionally modified by other compounds, we provide a detailed derivation in due course and refer to this non-linear

dependence by $F\left([(\text{GR-COR})_{2,\text{nu}}], [\text{COR}_\text{in}], [\text{GR}]\right)$ for now. Eventually, this yields the following ODE:

$$\frac{\mathrm{d}[\text{COR}_\text{in}]}{\mathrm{d}t} = k_\text{diff1}\left([\text{COR}_\text{ex}] - [\text{COR}_\text{in}]\right) - d_4\,[\text{COR}_\text{in}] + F\left([(\text{GR-COR})_{2,\text{nu}}], [\text{COR}_\text{in}], [\text{GR}]\right)\ . \tag{4}$$

The next step is the formation of the GR complex, which happens on a much faster timescale compared to the previously discussed molecular mechanisms. Hence, we may assume that the complex formation is in (quasi-)equilibrium, i.e.:

$$k_\text{GRC}\,[\text{GR-COR}] = [\text{COR}_\text{in}] \cdot [\text{GR}]\ , \tag{5}$$

with the dissociation constant k_GRC. The GR complex forms a homodimer, where we assume the dimerization to be in a (quasi-)equilibrium. By the proposed approach we consider the homodimer $(\text{GR-COR})_{2,\text{in}}$ as a separate species and thus as a monomer. Consequently, the concentration of the homodimer is given by:

$$k_\text{GRdim}\,[(\text{GR-COR})_{2,\text{in}}] = [\text{GR-COR}]^2\ , \tag{6}$$

where k_GRdim denotes the dissociation constant. The dimerized GR complex is transported to the nucleus, which we model by the following ODE:

$$\frac{\mathrm{d}[(\text{GR-COR})_{2,\text{nu}}]}{\mathrm{d}t} = k_\text{2in}\,[(\text{GR-COR})_{2,\text{in}}] - k_\text{2ex}\,[(\text{GR-COR})_{2,\text{nu}}] - d_9\,[(\text{GR-COR})_{2,\text{nu}}]\ . \tag{7}$$

The next ODE concerns the RNA sequence obtained when transcribing the GR gene. The transcription is determined by a basal rate v_7 and the catalysis by the GR complex with the maximal rate v_8 and the dissociation/Michaelis-Menten constant K_9. The RNA sequence is then transported to the cytoplasma (k_trs2) or degraded (d_{15}). The final ODE reads:

$$\frac{\mathrm{d}[\text{pmGR}]}{\mathrm{d}t} = -k_\text{trs2}\,[\text{pmGR}] + v_7 + \frac{v_8\,[(\text{GR-COR})_{2,\text{nu}}]}{K_9 + [(\text{GR-COR})_{2,\text{nu}}]} - d_{15}\,[\text{pmGR}]\ . \tag{8}$$

Consequently, the equation with respect to the messenger RNA mGR is given by:

$$\frac{\mathrm{d}[\text{mGR}]}{\mathrm{d}t} = \frac{V_\text{nu}}{V_\text{in}}\,k_\text{trs2}\,[\text{pmGR}] - d_{11}\,[\text{mGR}]\ . \tag{9}$$

The concentration of the receptor molecule GR is modulated by the translation of the respective mRNA and again by the non-linear dependence on the transport of the dimerized GR complex. Hence, we obtain the ODE:

$$\frac{\mathrm{d}[\text{GR}]}{\mathrm{d}t} = k_\text{tl2}\,[\text{mGR}] + F\left([(\text{GR-COR})_{2,\text{nu}}], [\text{COR}_\text{in}], [\text{GR}]\right) - d_8\,[\text{GR}]\ . \tag{10}$$

We now consider the fast feedback mechanism via the glucocorticoid membrane receptor. Concerning the production of the GPCR receptor we do not include regulation by means of other compounds. Therefore, it is admissible to provide only an abstract formulation, disregarding the processes on the genomic level. The ODE reads:

$$\frac{\mathrm{d}[\mathrm{GPCR}]}{\mathrm{d}t} = v_2 - d_6\,[\mathrm{GPCR}]\;, \tag{11}$$

where v_2 denotes a basal production rate and d_6 the degradation rate. With respect to the complex formation we can again assume a (quasi-)equilibrium, which is particularly justified as it has been experimentally observed that two ligands bind to the receptor and exhibit positive cooperativity (cf. [3]). We account for the positive cooperativity of the second cortisol molecule binding by a Hill factor. The dissociation constant of the complex is denoted by k_{GC2} and hence the algebraic relation reads:

$$k_{\mathrm{GC2}}\,[\mathrm{GPCR\text{-}(COR)_2}] = [\mathrm{COR_{ex}}]^2 \cdot [\mathrm{GPCR}]\;. \tag{12}$$

The next pathway we consider concerns the feedback mechanism related to CRH and the respective membrane receptor CRHR. Analogous to the ODE for the GPCR receptor, we use the following equation for the CRH receptor:

$$\frac{\mathrm{d}[\mathrm{CRHR}]}{\mathrm{d}t} = v_3 - d_7\,[\mathrm{CRHR}]\;, \tag{13}$$

with the basal expression rate v_3 and the degradation rate d_7. Concerning the complex formation we assume the following relation:

$$k_{\mathrm{CRC}}\,[\mathrm{CRHR\text{-}CRH}] = [\mathrm{CRH}] \cdot [\mathrm{CRHR}]\;, \tag{14}$$

where k_{CRC} is the respective dissociation constant. The binding of CRH to the receptor induces two signaling cascades regulating the release of ACTH and the production of certain transcription factors (summarized by TFs here). We start with the formulation of the equation for the transcription factors. A detailed modeling of the signaling cascade is beyond the scope of the model here. Consequently, we consider only the cytoplasmic reactions with respect to the TFs production. The stimulating effect by the signaling cascade is modeled by a standard saturation term, where we account for the fast dynamics of the signaling cascade by an additional modeling parameter h. Eventually, we have:

$$\frac{\mathrm{d}[\mathrm{TFs_{in}}]}{\mathrm{d}t} = \frac{V_{\mathrm{nu}}}{V_{\mathrm{in}}}\,k_{3\mathrm{ex}}\,[\mathrm{TFs_{nu}}] - k_{3\mathrm{in}}\,[\mathrm{TFs_{in}}] + \frac{v_4\,[\mathrm{CRHR\text{-}CRH}]^h}{K_6^h + [\mathrm{CRHR\text{-}CRH}]^h} - d_{12}\,[\mathrm{TFs_{in}}]\;. \tag{15}$$

The parameters $k_{3\mathrm{ex}}$ and $k_{3\mathrm{in}}$ concern the transport/diffusion of the transcription factors to the nucleus. v_4 indicates the limiting rate with respect to the stimulus of the CRHR-CRH complex and K_6 the sensitivity

to the stimulus. d_{12} is again a standard degradation rate. This leads to the ODE:

$$\frac{\mathrm{d}[\mathrm{TFs}_{\mathrm{nu}}]}{\mathrm{d}t} = k_{3\mathrm{in}}\,[\mathrm{TFs}_{\mathrm{in}}] - k_{3\mathrm{ex}}\,[\mathrm{TFs}_{\mathrm{nu}}] - d_{13}\,[\mathrm{TFs}_{\mathrm{nu}}]\;. \tag{16}$$

Concerning the regulation of the POMC gene via the dimerized GR-COR complex and the TFs transcription factors, it has been observed that these two types counteract via competitive inhibition. This yields the following equation for the transcribed RNA in the nucleus:

$$\frac{\mathrm{d}[\mathrm{pmPOMC}]}{\mathrm{d}t} = v_5 + \frac{v_6\,[\mathrm{TFs}_{\mathrm{nu}}]}{K_7\;(1 + [(\mathrm{GR\text{-}COR})_{2,\mathrm{nu}}]/K_8) + [\mathrm{TFs}_{\mathrm{nu}}]} - k_{\mathrm{trs1}}\,[\mathrm{pmPOMC}] - d_{14}\,[\mathrm{pmPOMC}]\;. \tag{17}$$

The transcription is determined by the basal rate v_5 and the limiting rate v_6 regarding the regulation by means of the different transcription factors. The dissociation/Michaelis-Menten constant K_7 represents the binding affinity of the TFs transcription factors to the respective DNA sites. K_8 denotes the dissociation/Michaelis-Menten constant with respect to the dimerized GR-COR complex. The transport of the RNA to the cytoplasm is then accounted for by the parameter k_{trs1}. This implies the following ODE for the mRNA in the cytoplasm:

$$\frac{\mathrm{d}[\mathrm{mPOMC}]}{\mathrm{d}t} = \frac{V_{\mathrm{nu}}}{V_{\mathrm{in}}}\,k_{\mathrm{trs1}}\,[\mathrm{pmPOMC}] - d_{10}\,[\mathrm{mPOMC}] \tag{18}$$

The modulation of the intracellular ACTH concentration depends on the translation and the cleavage of the POMC protein and its release from the vesicles, which is regulated by the feedback controls related to the two membrane receptors. Due to the fast response to the binding of the ligands by means of signaling cascades, we assume that the release of ACTH directly depends on the amount of formed receptor ligand complexes, where the catalysis of the CRH-receptor complex and the inhibitory effect of the cortisol-receptor complex are modeled as competitive inhibition as suggested in [4]. This yields the following ODE:

$$\frac{\mathrm{d}[\mathrm{ACTH}_{\mathrm{in}}]}{\mathrm{d}t} = k_{\mathrm{tl1}}\,[\mathrm{mPOMC}] - d_5[\mathrm{ACTH}_{\mathrm{in}}] - \frac{k_{1\mathrm{ex}}\,[\mathrm{CRHR\text{-}CRH}]\cdot[\mathrm{ACTH}_{\mathrm{in}}]}{K_4\;(1 + [\mathrm{GPCR\text{-}(COR)_2}]/K_5) + [\mathrm{CRHR\text{-}CRH}]}\;. \tag{19}$$

The parameter k_{tl1} describes the translation rate of the POMC mRNA, where we additionally incorporate the cleavage. d_5 is the degradation rate of $\mathrm{ACTH}_{\mathrm{in}}$. In accordance with the competitive inhibition, K_4 and K_5 denote the sensitivity with respect to the two counteracting signals. Based on (19) the ODE with respect to the extracellular ACTH concentration immediately follows:

$$\frac{\mathrm{d}[\mathrm{ACTH}_{\mathrm{ex}}]}{\mathrm{d}t} = \frac{V_{\mathrm{in}}}{V_{\mathrm{ex}}}\,\frac{k_{1\mathrm{ex}}\,[\mathrm{CRHR\text{-}CRH}]\cdot[\mathrm{ACTH}_{\mathrm{in}}]}{K_4\;(1 + [\mathrm{GPCR\text{-}(COR)_2}]/K_5) + [\mathrm{CRHR\text{-}CRH}]} - d_3\,[\mathrm{ACTH}_{\mathrm{ex}}]\;. \tag{20}$$

The last open relation is the dependence of the cortisol and GR concentration on the diffusion/transport of the dimerized glucocorticoid receptor complex into the nucleus. Since we do not explicitly model the complex or the resulting homodimer, we obtain a non-standard non-linear relation, which we derive in the following. This relation directly follows from the already made assumptions and does not assume any further simplifications. From the equilibrium assumptions (5) and (6) we obtain:

$$k_{\mathrm{GRdim}} \frac{\mathrm{d}[(\text{GR-COR})_{2,\mathrm{in}}]}{\mathrm{d}t} = 2\,[\text{GR-COR}]\frac{\mathrm{d}[\text{GR-COR}]}{\mathrm{d}t}\ , \tag{21}$$

$$k_{\mathrm{GRC}} \frac{\mathrm{d}[\text{GR-COR}]}{\mathrm{d}t} = \frac{\mathrm{d}[\mathrm{COR_{in}}]}{\mathrm{d}t}[\mathrm{GR}] + [\mathrm{COR_{in}}]\frac{\mathrm{d}[\mathrm{GR}]}{\mathrm{d}t}\ , \tag{22}$$

by differentiating. As we are interested in the impact of the transport/diffusion of $(\text{GR-COR})_2$ into the nucleus we may write:

$$\frac{\mathrm{d}[(\text{GR-COR})_{2,\mathrm{in}}]}{\mathrm{d}t} = \frac{V_{\mathrm{nu}}}{V_{\mathrm{in}}}\left(k_{2\mathrm{ex}}\,[(\text{GR-COR})_{2,\,\mathrm{nu}}] - k_{2\mathrm{in}}\,[(\text{GR-COR})_{2,\mathrm{in}}]\right)\ , \tag{23}$$

i.e. the right hand side describes the impact of the translocation on the concentration of $(\text{GR-COR})_2$ over time. We reasonably may assume that the cell is neither depleted from cortisol nor GR. If so, also no complex can form and we have no transport/diffusion into the nucleus. Hence, we can conclude from relation (21):

$$\frac{\mathrm{d}[\text{GR-COR}]}{\mathrm{d}t} = k_{\mathrm{GRdim}}\,\frac{1}{2}\,\frac{V_{\mathrm{nu}}}{V_{\mathrm{in}}}\,\frac{k_{2\mathrm{ex}}\,[(\text{GR-COR})_{2,\,\mathrm{nu}}] - k_{2\mathrm{in}}\,[(\text{GR-COR})_{2,\mathrm{in}}]}{[\text{GR-COR}]}\ , \tag{24}$$

and equation (22) yields:

$$\bar{k}\,k_{\mathrm{GRC}}\,k_{\mathrm{GRdim}}\,\frac{k_{2\mathrm{ex}}\,[(\text{GR-COR})_{2,\,\mathrm{nu}}] - k_{2\mathrm{in}}\,[(\text{GR-COR})_{2,\,\mathrm{in}}]}{[\text{GR-COR}]} = \frac{\mathrm{d}[\mathrm{COR_{in}}]}{\mathrm{d}t}[\mathrm{GR}] + [\mathrm{COR_{in}}]\frac{\mathrm{d}[\mathrm{GR}]}{\mathrm{d}t}\ , \tag{25}$$

with $\bar{k} := \frac{V_{\mathrm{nu}}}{2\,V_{\mathrm{in}}}$. As the dimerized glucocorticoid receptor complex consists of two cortisol and two GR receptors, we obtain:

$$\frac{\mathrm{d}[\mathrm{COR_{in}}]}{\mathrm{d}t} = \frac{\mathrm{d}[\mathrm{GR}]}{\mathrm{d}t}\ , \tag{26}$$

with respect to the influence of the transport/diffusion into the nucleus, i.e. ignoring any degradation or diffusion out of the cell for now. Reformulating $[(\text{GR-COR})_{2,\mathrm{in}}]$ and $[\text{GR-COR}]$ by means of the assumptions (5) and (6) in equation (25) consequently yields the rate of change for COR and GR concerning the diffusion/transport of the dimerized glucocorticoid receptor complex into the nucleus. That is, the function F is given by:

$$F\left([(\text{GR-COR})_{2,\mathrm{nu}}], [\mathrm{COR_{in}}], [\mathrm{GR}]\right) := \bar{k}\,\left(\frac{k_{2\mathrm{ex}}\,k_{\mathrm{GRC}}^2\,k_{\mathrm{GRdim}}\,[(\text{GR-COR})_{2,\mathrm{nu}}]}{[\mathrm{COR_{in}}]^2\cdot[\mathrm{GR}] + [\mathrm{COR_{in}}]\cdot[\mathrm{GR}]^2} - \frac{k_{2\mathrm{in}}\,[\mathrm{COR_{in}}]\cdot[\mathrm{GR}]}{([\mathrm{COR_{in}}] + [\mathrm{GR}])}\right)\ , \tag{27}$$

with $\bar{k} = \frac{V_{\text{nu}}}{2\,V_{\text{in}}}$.

References

1. Zarzer CA: **Sparsity Enforcing Regularization on the ℓ_p-Scale with $p < 1$.** *PhD thesis*, Johannes Kepler University Linz 2012.

2. Gupta S, Aslakson E, Gurbaxani BM, Vernon SD: **Inclusion of the glucocorticoid receptor in a hypothalamic pituitary adrenal axis model reveals bistability**. *Theor Biol Med Model* 2007, **4**:8.

3. Maier C, Runzler D, Schindelar J, Grabner G, Waldhausl W, Kohler G, Luger A: **G-protein-coupled glucocorticoid receptors on the pituitary cell membrane.** *J Cell Sci* 2005, **118**(Pt 15):3353–61.

4. Lu L, Suzuki T, Yoshikawa Y, Murakami O, Miki Y, Moriya T, Bassett MH, Rainey WE, Hayashi Y, Sasano H: **Nur-related factor 1 and nerve growth factor-induced clone B in human adrenal cortex and its disorders**. *J. Clin. Endocrinol. Metab.* 2004, **89**:4113–4118.
